# ‘Mind the gaps’: the accessibility and implementation of an effective depression relapse prevention programme in UK NHS services: learning from mindfulness-based cognitive therapy through a mixed-methods study

**DOI:** 10.1136/bmjopen-2018-026244

**Published:** 2019-09-08

**Authors:** Jo Rycroft-Malone, Felix Gradinger, Heledd Owen Griffiths, Rob Anderson, Rebecca Susan Crane, Andy Gibson, Stewart W Mercer, Willem Kuyken

**Affiliations:** 1 School of Health Sciences, Bangor University, Bangor, UK; 2 Institute of Health Research, University of Exeter, Exeter, UK; 3 Peninsula Technology Assessment Group (PenTAG), University of Exeter, Exeter, UK; 4 School of Psychology, Bangor University, Bangor, UK; 5 Health and Social Sciences, University of the West of England, Bristol, UK; 6 Institute of Health and Wellbeing, University of Glasgow, Glasgow, UK; 7 Psychiatry, University of Oxford, Oxford, UK

**Keywords:** implementation, implementation science, psychological therapy, mindfulness, mindfulness-based cognitive therapy, depression, PARIHS

## Abstract

**Objectives:**

Mindfulness-based cognitive therapy (MBCT) is an evidence-based approach for people at risk of depressive relapse to support their long-term recovery. However, despite its inclusion in guidelines, there is an ‘implementation cliff’. The study objective was to develop a better explanation of what facilitates MBCT implementation.

**Setting:**

UK primary and secondary care mental health services.

**Design, participants and methods:**

A national two-phase, multi-method qualitative study was conducted, which was conceptually underpinned by the Promoting Action on Research Implementation in Health Services framework. Phase I involved interviews with stakeholders from 40 service providers about current provision of MBCT. Phase II involved 10 purposively sampled case studies to obtain a more detailed understanding of MBCT implementation. Data were analysed using adapted framework analysis, refined through stakeholder consultation.

**Results:**

Access to MBCT is variable across the UK services. Where available, services have adapted MBCT to fit their context by integrating it into their care pathways. Evidence was often important to implementation but took different forms: the NICE depression guideline, audits, evaluations, first person accounts, experiential taster sessions and pilots. These were used to build a platform from which to develop MBCT services. The most important aspect of facilitation was the central role of the MBCT implementers. These were generally self-designated individuals who ‘championed’ grass-roots implementation. Our explanatory framework mapped out a prototypical implementation journey, often over many years with a balance of bottom-up and top-down factors influencing the fit of MBCT into service pathways. ‘Pivot points’ in the implementation journey provided windows of either challenge or opportunity.

**Conclusions:**

This is one of the largest systematic studies of the implementation of a psychological therapy. While access to MBCT across the UK is improving, it remains patchy. The resultant explanatory framework about MBCT implementation provides a heuristic that informed an implementation resource.

**Strengths and limitations of this study**

- This is one of the largest studies of what facilitates implementation of a psychological therapy within a public health service.
- Data collection and analysis were theoretically driven and systematically conducted, including the perspectives of the research team, service user groups and key stakeholder groups.
- The study is limited to a particular treatment (mindfulness-based cognitive therapy) and to the UK healthcare service.

## Background

Depression is one of the most common mental health problems, affecting as many as one in five people in their lifetime.^1^ It often runs a recurrent lifetime course, is often comorbid with other physical and mental health problems and is a major cost to society.^2–4^ While 23% of the total burden of disease is attributable to mental health problems, typically nations spend a much smaller fraction of health expenditure on mental health.^5^

The last 50 years has seen a transformation in mental health with the development of a range of psychological treatments for depression that are effective and cost effective^6 7^; significant advances in understanding and changes in public attitudes^8^ and a recognition that upstream preventative interventions may be a promising way of addressing this major public health challenge. That is to say, the substantial health burden attributable to depression could be offset through making evidence-based interventions that prevent depressive relapse among people at risk of recurrent depression more accessible.^9^ Health economists have made the case that the modest cost of effective psychological treatments would be repaid in enhanced productivity, tax receipts and reduced disability benefits,^6 10^ in addition to improvements in quality of life.

Mindfulness-based cognitive therapy (MBCT) was developed as a group-based psychosocial approach to help people at risk of depressive relapse learn skills to prevent future episodes. The developers have written session-by-session guides for MBCT teachers^11^ and patients.^12^ MBCT integrates the basic structure of mindfulness-based stress reduction^13^ with elements from cognitive-behavioural therapy.^14^ It is taught in 8 weekly 2-hour-long classes of 8–15 people. MBCT is psychoeducational and is largely based on experiential learning; which is why MBCT teachers are normally called ‘teachers’ rather than ‘therapists’. Its effectiveness has been demonstrated in numerous randomised controlled trials.^15^ National treatment guideline groups have recommended it for depression relapse prevention since 2004.^16 17^ Stakeholders, patient groups and an All Party Parliamentary Group in the UK have called for it to be made more readily available in the National Health Service.^18^ Our literature review and feasibility work suggest that while there are exemplars of effective, sustainable MBCT implementation, access is inequitably distributed.^19^ A 2015 workforce census suggests that the majority of National Health Service (NHS) Trusts in England have no one who is trained to deliver MBCT.^20^ There is no systematic understanding about why MBCT is not being more widely implemented within health services. This study aimed to fill that gap.

The aims of this study were to

- Scope services and develop an understanding of the perceived benefits and costs of embedding MBCT in mental health services.
- Explore facilitators and barriers to MBCT implementation.
- Articulate the critical success factors for enhanced accessibility and the routine and successful use of MBCT.
- Synthesise the evidence from these data sources and develop an explanatory framework of MBCT implementation.

## Methods

### Design

We conducted a two-phase qualitative, exploratory and explanatory study, which was conceptually underpinned by the Promoting Action on Research Implementation in Health Services (PARIHS) framework.^21 22^ PARIHS was developed to represent the complexities involved in the successful implementation of evidence into practice. Successful implementation (SI) is represented as a function (f) of the nature and type of evidence (E) being implemented, the qualities of the context (C) in which it is being implemented and the process of facilitation (F) (SI=f(E,C,F). The framework was used as a heuristic to inform the development of interview and observation schedules, and in the analysis process.

The study protocol has been previously published^23^ and the methods, including the interview schedules described fully in the final report.^19^

### Phase I: interview study

To obtain an overview of whether and how MBCT is being delivered in the four countries of the UK, and to provide an overview of the factors that have facilitated and hindered implementation, we conducted 68 semi-structured interviews with participants from 40 service providers across the UK.

First, we conducted interviews with people who were known to have a stake in MBCT delivery through existing networks and snow-balling. We included 27 MBCT teachers and 20 clinicians in a management role. We sampled interviewees to ensure at least one key informant from each stakeholder group per site. We explicitly tried to recruit people with a diversity of perspectives and backgrounds. Second, to ensure representativeness, we created a sampling pool of 91 candidates, which included 39 managers, 23 commissioners, 16 service users and 13 referrers. We then randomly sampled (using a simple random numbers block sampling method) across this participant pool and interviewed seven managers, four commissioners, five referrers and five service users.

### Phase II: case studies

Building from Phase I’s overview, case studies were aimed to develop a more in-depth and contextually embedded understanding of MBCT implementation.^24^ A case study is particularly appropriate for when ‘a how or why question is being asked about a contemporary set of events over which the investigator has little or no control’.^25^ Ten cases were purposively sampled to ensure UK’s four countries' representation and the level of embeddedness of MBCT provision (four fully embedded, four partially embedded and two scarce/no implementation).^19 23^ The level of embeddedness was determined based on a set of criteria derived from national guidance and best practice about the features that should be in place to deliver an appropriate MBCT service.

### Data collection

See table 1 for a summary of the case study sample. Data were collected between 2013 and 2015.

**Table 1:** Case study data collection

Site	Total interviews	MBCT teacher	Manager	Referrer/GP/ Commissioner	Service user	Total observations	Total documents
Elm	20	6	6	2	6	7	6
Pine	17	5	8	2	2	2	6
Oak	20	6	9	2	3	4	6
Mangrove	7	3	2	2	0	0	5
Beech	9	4	4	1	0	1	3
Juniper	10	6	3	0	1	0	3
Bamboo	14	7	3	4	0	1	5
Birch	11	7	4	0	0	1	6
Wisteria	7	3	3	0	1	1	4
Hazel	12	7	4	1	0	0	3
Total	127	54	46	14	13	17	47

GP, general practitioner; MBCT, mindfulness-based cognitive therapy

#### Semi-structured interviews

Schedules were initially informed by Phase I findings; as data collection progressed, we revised these to build up an explanation across cases. Interviews were audio recorded and transcribed in full.

#### Observations

Naturally occurring meetings and events including supervision, special interest groups, service user sessions and teacher training sessions, which complemented interview data. Data were recorded as field notes.

#### Documents

To help build an explanation of MBCT implementation within cases, relevant documents such as mental health strategies, training pathways and funding cases were collected alongside publicly available contextual information such as CQC ratings, trust websites, demographics for socioeconomics, ethnicity and mental health metrics.

### Data analysis

Data were managed in Atlas.ti,^26^ Excel and Word. In both phases, data were analysed using an iterative, and combined inductive and deductive thematic analysis process.^19 27^ In Phase I, we built a description of MBCT implementation by coding interview data using the main elements of the PARIHS framework: evidence, context and facilitation. This provided a conceptual map, which was used to move onto Phase II data collection. In Phase II, we developed a coding framework inductively from data collected in the early case study sites. Each data source was considered separately, before building case summaries by combining coding from all data sources. A cross case explanation was built up inductively from the case summaries using a pattern matching logic. The face validity of the resultant explanatory framework was checked through consultation with a range of stakeholders in three workshops.

### Patient and public involvement

Our approach to patient and public involvement (PPI) was described in full in our published study protocol.^23^ PPI involvement comprised a panel convened by one of the authors (AG), MBCT advocates and ‘critical friends’. They met through the life of the project, participated in the Project Advisory Group, contributed to the study protocol, commented on study materials, reviewed and commented on the Phase I and II analyses and co-ran some of the dissemination workshops.

## Results

We report the findings from Phase I and II as a whole, moving from a description of the main facilitators of MBCT implementation, to a more in-depth reporting of the features of the explanatory framework.

### Facilitators of MBCT implementation in the UK NHS

The main elements of the PARIHS framework, evidence, context and facilitation, capture the main features of MBCT implementation. Perhaps, unsurprisingly, MBCT teachers were able to share more information about activities, facilitators and barriers related to getting MBCT used in practice.

*Evidence* could take different forms including evidence-based clinical guidelines, audits, evaluations, experiential first-person accounts and pilots that were used to build a platform from which to evolve and develop local MBCT services.

National clinical guidelines recommending MBCT helped give credibility, secure funding and ‘really sort of just opened the door I would say. I think without… just having MBCT in the NICE guidelines, just creates a legitimacy in people’s minds that… I don’t think we or others could have got nearly as far with this, you know without that, and without the other research that’s you know mushrooming now in mindfulness, and that we can refer to.’ (MBCT Teacher).

However, guidelines could be a ‘double-edged sword’ (MBCT Teacher) as their prescriptive nature could be at odds with the need for adapting MBCT to particular service and service user demands. There was evidence that effective implementers made adaptation and formally evaluated these adaptations within the service to establish their acceptability and effectiveness. For example, this might involve broadening the inclusion criteria for MBCT and establishing whether this compromised MBCT’s acceptability and effectiveness.

Participants reported using other types of evidence as facilitators, including clinical judgement: ‘there’s a way of having clinical judgement and being able to say why it’s useful, justify your decision, justify inviting somebody to the group’ (MBCT Teacher). Client feedback, both MBCT course participants and teachers, was another important type of evidence, with this being collected anecdotally but also through formal evaluation of MBCT courses.

*Context* was a powerful facilitator of MBCT implementation. The context of NHS services for MBCT’s particular aims, ethos and methods was key. MBCT is a prevention programme in which participants learn mindfulness and cognitive behavioural therapy skills to support their long-term recovery.^28 29^ A dominant theme was the tension between the culture of the health service and MBCT’s ethos. One MBCT teacher described it as follows:

quite a struggle…to deliver the program in a mindful way, so like all the administration around it and so on, because the culture of IAPT (Improving Access to Psychological Therapies) and primary care therapy… tends to be therapy on roller skates, that’s the whole culture, its bums on seats, quickly in, quickly out, have we got to recovery, off we go, quick team meeting, you know, rush, rush, rush.

The NHS was described as focused primarily on physical health, using a medical model which includes treatment to recovery, fast-paced, ever-changing and target-driven. An MBCT teacher described how this created

…a real tension between integrity and fast implementation. It was quite difficult to hold the line there, and really not be drawn into providing a level of service that just didn’t seem appropriate.

These tensions created barriers for MBCT implementation. However, some MBCT teachers used ‘the chaos to embed MBCT’, enabling implementation to go forward ‘under the radar’. They were able to reframe these barriers as opportunities for working creatively in ways that might not otherwise have been possible.

MBCT was often implemented by securing resources informally. This was a noticeable feature of both the fragility of implementation and the resourcefulness of implementers. Human resources normally comprised a single or small number of champions teaching and implementing MBCT in addition to their existing roles and responsibilities. ‘If I was to leave tomorrow, essentially that mindfulness service would die with me’ (MBCT Teacher, South). There were variants on this theme, for example,

The way we’ve organised it so far is to identify a lead practitioner within the XXXX (Trust) who has this as part of her remit, XXXX, but she’s had this sort of crowbarred into her ordinary job description and like a lot of psychotherapy training and treatments sometimes it just has to run on enthusiasm rather than organisation … oomph if you know what I mean and some of the personal commitment that people bring is what keeps it going (Head of Psychological Therapies).

MBCT implementation requires financial resources, for example, for the training and supervision of MBCT teachers; however, these were rarely available. In many cases, MBCT teachers had paid for their own training and supervision because of their professional and personal commitment to MBCT. In some cases, they had personally resourced the classes in terms of venues, mindfulness recordings and yoga mats. These resources alongside the time and finances to secure them was a major barrier to implementation. The resourcefulness of MBCT teachers was common:

Money, money, money, money, oh my god, one year, in order to get CDs for the course, I had to dig up all the strawberry plants out of my garden, and sell them, and colleagues helped, and we had a cake and plant sale, and raised £400 and then bought our CDs for the courses. Getting mats, I’ve had to beg and borrow every March from Senior Management (MBCT teacher).

In terms of *facilitation*, the most notable and critical finding relevant to implementation was the role of champions and championing. MBCT implementation was invariably driven by ‘passionate’ champions ‘willing to go the extra mile’. However, they often developed particular implementation skills and were well placed within their services to effect change. Typically, champions were MBCT teachers, but sometimes they were service managers or people in receipt of services:

We created a new job that is explicitly the specialist care pathway lead for MBCT…it’s easier for me to get these things done because of the position I hold and it was easier for me to create a post and sort of empower this individual to do this project (Manager).

There were also exemplars of service users being engaged in implementation, in a variety of ways, including developing course materials.

### The how and why of MBCT implementation: an explanatory framework

**Figure 1:**
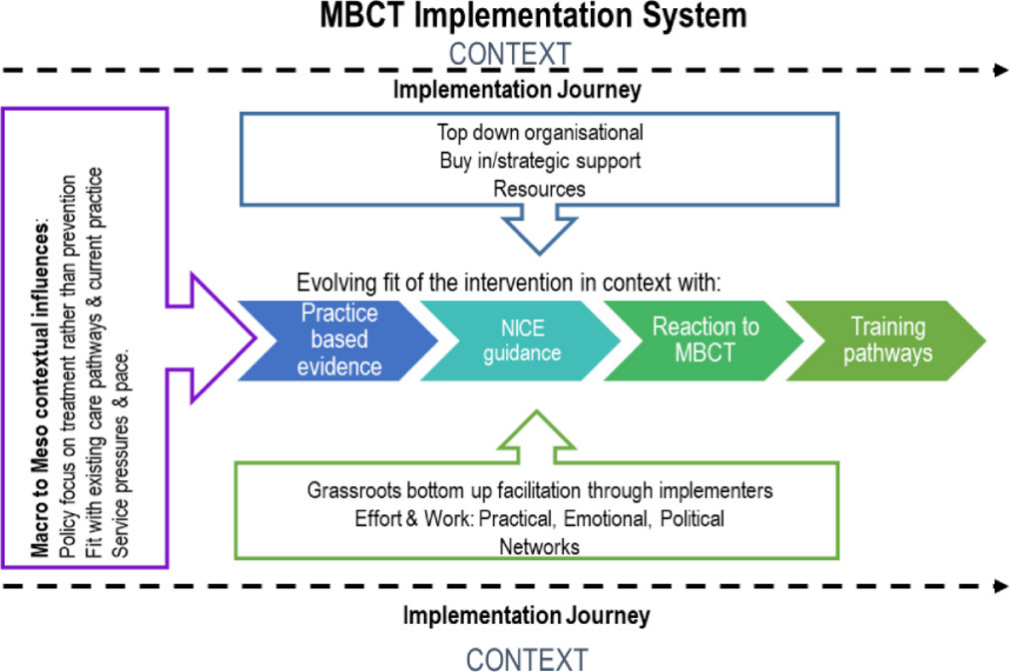
Explanatory framework. MBCT, mindfulness-based cognitive therapy; NICE, National Institute for Health and Care Excellence.

This explanatory framework sets out the critical success factors for MBCT implementation, how they interact with one another and how this evolves over time. It proposes an overarching metaphor ‘the implementation journey’ and six inter-dependant explanations that constitute the ‘active ingredients’ of an implementation journey (see figure 1). While this representation loses some of the particularities of the implementation story of each case, it provides a theoretically transferable and overarching account to explain the how and why of MBCT implementation.

#### The implementation journey: a metaphor

Each implementation journey had a starting point, borne out of the particular conditions within the site. In some cases, implementation started before the 2008 financial crisis when resources were more readily accessible. In others, it was the launch of the England-wide Improving Access to Psychological Therapies programme with its focus on implementing evidence-based psychological treatments.^30^ In others, a shift in service ethos to greater focus on well-being and/or recovery seeded MBCT implementation. The prototypical journey always started with one or more MBCT champions engaging in extensive grass roots, bottom-up efforts, building momentum and support systems. The more successful implementation had more than one implementer, often at different levels in the organisation, with complementary skills aligned around the same agenda. While implementation typically started grass roots and bottom-up, over time, sometimes years, this would become integrated with top-down organisational commitment and investment. Moreover, in the most sustainable implementation journeys we saw evidence of active succession planning. Finally, each case study had key ‘pivot points’ in the journey which provided windows of challenge and opportunity.

The six inter-dependant elements that constitute the implementation journey are MBCT implementers, context, fit, training and supervision, top-down and bottom-up and pivot points.

##### MBCT implementers

MBCT implementation relied on the presence of at least one person who drove and led implementation—‘the implementer’. Implementation was more successful in some sites than others because of a combination of the implementer’s characteristics, skills and experience, the networks s/he created, or was part of, and the context in which they were working. Implementers were typically self-designated, skilled individuals who ‘championed’ MBCT implementation bottom-up, and over time, mobilised top-down organisational support.

Effective implementers demonstrated particular characteristics and skills.

It’s generally being conducted by enthusiasts…if you have experienced enthusiastic, committed, well trained, clever people, do almost anything, you know it’s gonna have a good outcome (Associate Medical Director Mental Health).

The following characteristics and skills were critical: personal interest and commitment, confidence, passion and dedication, communication skills (eg, diplomacy and adapting language for different stakeholders), business sense (eg, ‘selling’) and competency in research and evaluation (eg, evaluation and demonstrating impact).

Effective implementers organised a range of activities to support implementation. Key activities offered to support implementation were reported in both phases. However, in the case studies, we saw that the range of activities offered corresponded to the degree of MBCT service development, with more activities corresponding to more successful implementation effort.

Activities included taster sessions so stakeholders such as referrers, commissioners and managers could ‘experience it and appreciate it’ (Clinical Lead); integrating MBCT into the staff well-being services, offering mindfulness classes for staff; developing resources based on service aims and targets, evaluation ‘hard science and testimonials’ (Trainer) to reach ‘hearts and minds’ (Clinical Lead); and pilots and audits to generate a case based on the need for and potential impact of MBCT.

Implementers created informal and formal networks to share issues and expertise, learn and generate solutions to problems. Typically, these started as small, tight networks around the implementer (eg, local MBCT interest group), but as the implementation journey progressed these extended across the service to include a broader set of stakeholders (commissioners, managers, service users) as well as external networks of regional and national implementers, training and supervision networks and research and evaluation networks. In more established sites, there was evidence that a greater mix of formal and informal networks had evolved over time to meet key functions (eg, supervision, training, governance and research). There was evidence that successful implementation was associated with links with Universities, for example, to support research, evaluation and training.

The degree of implementation (ie, more to less embedded) was a function of the implementer’s characteristics, skills and experience, the activities and networks s/he generated, their position in the organisation and the context, to which we turn next.

##### Context

Implementation journeys took place in an ever-changing context at different levels, from national mental health policies, broader national and regional health service priorities, and the local service priorities, ways of working and culture. These contextual factors were more or less evident in all case studies. An accumulation of more facilitators than barriers and an alignment of the facilitators were associated with implementation:

I think we’ve been very lucky in this (site) that the number of elements combining to make it more possible…having a Chief Exec that was open to it, having a few people in the organisation that were keen for it to happen, a couple of consultants, [lead for depression service, academic] helped to get it started… They’re all at different levels in the organisation but they’ve all got influence so I think that creates an environment in which it can happen…if you’ve got people in the organisation saying ‘yes we want this to happen’ this enables the people lower down (Implementer).

A misalignment, particularly between middle and senior managers, presented significant challenges to implementers. For example, one implementer described how a service redesign meant that s/he was asked: ‘to put all my training activities on hold and to not provide any further mindfulness training… I was told to freeze all of my training activities and I was diverted to clearing the waiting list for individual therapy.’

Service pressures such as staff workload, turnover of work force, including managers, limited resources and working culture pose a challenging implementation context. A CEO put it this way:

we are struggling with year on year of historic disinvestment with escalating activity pressures due to the changing nature of urban boroughs, problems with staff, recruitment in terms of quality and all of those can add up to a pretty difficult cocktail when you’re trying to transform the way in which you work with people and do business.

##### Fit

Successful and sustainable implementation of MBCT was dependent on the alignment between the intervention and NHS context, existing local service strategy, priorities and pathways, and efforts to adapt and make it fit. Resilient implementation required creative and flexible adaptation to fit with other service initiatives, management interests, resources and the ethos or culture of the service (eg, medical model vs well-being vs recovery-oriented). Successful implementation was characterised by recognition of national and service performance targets, integration with extant service care pathways, alignment with senior managers’ and service users’ needs and interests and the prevailing organisational culture.

In some cases, there were mismatches, for example, a reorganisation that focused solely on core services to make savings or a focus on prioritised targets around acute and urgent treatment. Across the implementation journey, there was a need for fit to be continually re-evaluated and adapted, often with early grass-roots implementation being ‘under the radar’ and later implementation being more fully integrated at each level, local, regional and national.

##### Training and supervision

The existence and quality of training and supervision was a function of the level of strategic priority and subsequent investment placed on MBCT implementation within an organisation. There was typically a tension between ‘gold standard’ and ‘good enough’ MBCT teacher training models. Our data showed a mixed picture of training and supervision arrangements, based on an adaptation to local need, resource and capacity. One site that had created an internal ‘apprenticeship model’, others had commissioned in supervision and training from training centres, while others had ad hoc or no arrangements for supervision and training. For the most part, the UK Good Practice Guidelines for MBCT supervision and training^31^ provided guidance for many sites, but also created a tension between marrying up ‘the gold standard mindfulness training that our local centre provides, versus working out the politics of financing all of that and for us to be happy with an imperfect but nevertheless good enough level of provision’ (Head of Psychological Therapies). In one site, the Mindfulness Clinical Lead was asked, ‘is there a light version of MBCT,’ ‘is there something that’s shorter, less intensive, fewer hours and more easily provided without such stringent guidelines?’ And I said well I said there’s lots of other people doing that, but I said *‘*no, we’re an NHS provider service, we provide as per the NICE guidelines and we’re working with people who have mental health problems and deserve the evidence-based intervention…. So I said I will not support the trust in delivering a light version of MBCT, so no.’ The service was withdrawn.

The most embedded MBCT service had made a strategic investment in MBCT training and supervision, with a dedicated training lead role and internal apprenticeship training. This created a ‘sense of people feeling confident in the quality of the training that they feel able to train in ways and feel safe*’* (Research lead). In other sites, training had been ‘on a shoe string’ and/or staff paid for it out of personal resources and in their own time.

##### Top-down and bottom-up

Implementation of MBCT was driven by a combination of bottom-up activity and top-down support and investment, the effect of which was enhanced by middle management and clinician buy-in. However, the data were densely populated with examples of ‘bottom–up’ or grass-roots facilitation, involving champions raising awareness, building networks, securing MBCT teacher training and supervision. Only a small minority of examples were of ‘top–down’ facilitation, where service commissioners or managers instigated and drove implementation, although there were a few notable exceptions. For example, Scotland had introduced a nation-wide top-down implementation of MBCT through a training programme of mindfulness-based teachers in the NHS, although with limited funding which reduced over time.

The prototypical implementation journeys thus started with bottom-up grass roots led by a skilful, resourceful mindfulness practitioner who built formal and informal networks. However, for MBCT to become an embedded sustainable part of the service at a certain stage in the implementation journey, it needs top-down support and investment. This enabled MBCT services to become part of the service’s overarching strategy, engage a wider array of key stakeholders, secure essential resources and establish structures and processes that embedded MBCT within the governance and care pathways of the service. Without this top-down buy-in and support, implementation tended to be limited in scope and fragile over time. There was evidence to suggest that middle management was particularly key in either enabling or hindering sustainable implementation.

##### Pivot points

**Figure 2:**
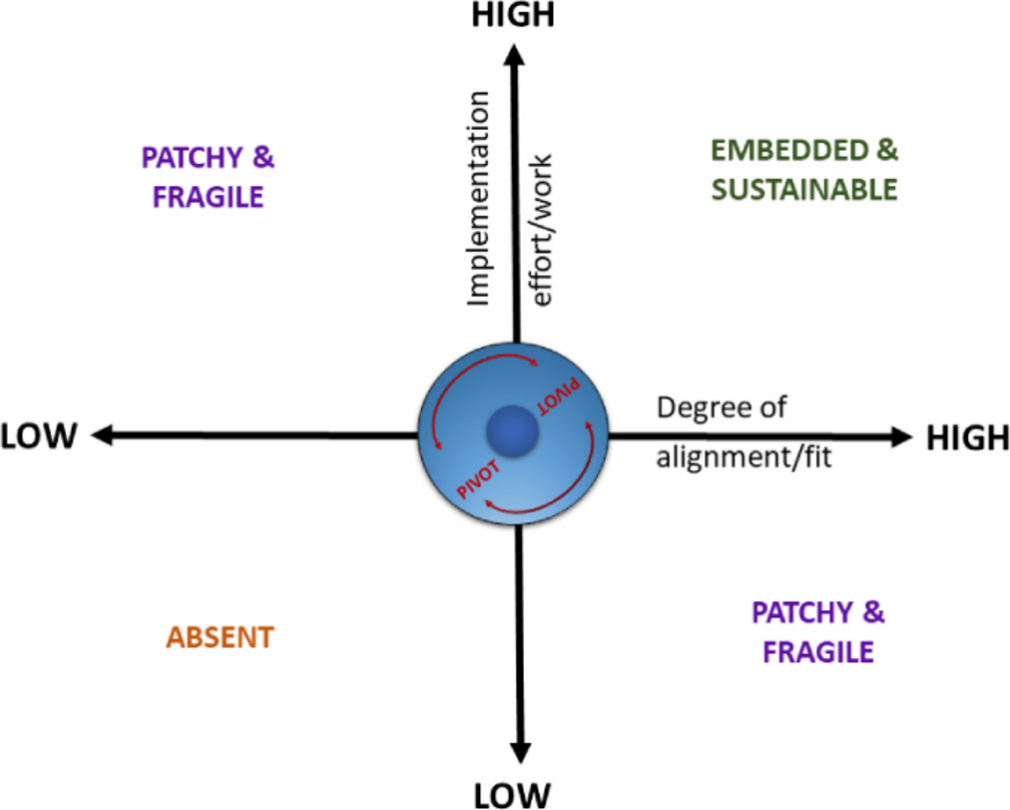
Implementation pivot points.

A combination of factors led to pivot points, or key moments, in a site’s implementation journey, which were critical junctures where implementation accelerated, was impeded or stalled (figure 2). While pivot points were different in each site, both in terms of timing and combination of factors, they shared some common features. These included, the coming together of adequate resources, an organisational structure (including investment) to deliver MBCT within services, a critical mass of MBCT teachers, the development (and persuasion) of a compelling case informed by different forms of evidence, nationally badged guidance alongside evidence of local impact and making visible the potential of MBCT through major events. For example, in 2015, the Mindful Nation UK All Party Parliamentary Group report^18^ was published, including specific recommendations with respect to MBCT and the NHS. This publication alongside a range of stakeholder events is an example of a major event that contributed to these pivot points within services. The coming together of these factors to facilitate a better fit (high–low) of MBCT into service provision was a function of the amount (high–low) of effort or work of local implementers and the contexts of implementation. The effort and work were correlated with the degree of success of implementation—patchy, fragile, absent and embedded.

## Discussion

A 2012 British Medical Journal editorial set out key remaining questions about MBCT and recurrent depression, and included the questions: ‘What are the facilitators and barriers to implementation of NICE’s recommendations for MBCT in the UK’s health services? Can this knowledge be used to develop an Implementation Plan for introducing MBCT consistently into NHS service delivery?’^32^ This research has gone a long way to answering these questions. Moreover, it resonates closely with a recent multiple case study exploration of implementing mindfulness training in schools, which identified four cornerstones of implementation: people, journey, resources and perceptions of mindfulness.^33^ Moreover, it resonates with the broader implementation science literature, especially the PARIHS framework in which champions are consistently found to be key in facilitating new evidence into practice, through skilful alignment of an intervention with the local context.^21 32 33^

The context for MBCT implementation operated at multiple levels, from national policies, regional priorities, to service culture and specifications. Sustainable implementation was characterised by implementers who could recognise these contextual factors and the synergies and tensions they create, and find a way to integrate and ‘fit’ MBCT into the prevailing context. Significantly, this meant that often MBCT was implemented in ways other than those recommended by NICE and sometimes that the intervention itself was adapted. While this supported implementation, its effects on effectiveness are unknown. A recent pragmatic study of MBCT’s effectiveness in NHS services sampled from some of our case study sites suggested that MBCT’s acceptability and outcomes were comparable when benchmarked against outcomes in randomised trials.^34^ Implementation literature^35 36^ suggests a dynamic interplay between an intervention, its implementation and context. An aspect of context in one area may be a facilitator in one case but a barrier in another; moreover, implementing an intervention like MBCT may change context and culture over time.

Evidence took various forms (from national clinician guidelines to service user testimonies) and was used in implementation in different ways at different times. This mirrors both the PARIHS framework^37–40^ and evidence from implementation and innovation more broadly.^41^ Evidence needed to be transformed and particularised to make it relevant and applicable. This is shaped by the context (eg, setting service priorities or piloting a new service) and the group doing this individualisation (eg, service users or managers). Facilitation was driven by committed, skilled, usually self-designated implementers, who championed MBCT, initially as a grass roots, bottom-up drive was writ large across our data. As already noted, human change agents are a theme common to successful implementation in healthcare.^42^ The centrality of champions in MBCT implementation is both an opportunity (eg, identifying, resourcing and supporting champions) and potential risk (eg, champions who become over-extended, burn out and/or leave).

Our explanatory framework suggests a prototypical implementation journey, often over many years (figure 1). The framework provides clear ideas for how services could implement MBCT at different stages in the implementation journey. Using the explanatory framework and the data corpus, we developed an online resource designed to support implementation.^43^ This is primarily a practical resource, but could also be the basis for future research evaluating the effectiveness of the explanatory framework in supporting implementation.

Successful implementation is likely to be supported by attending to the common features we observed across our data. The implementer championing MBCT is key. S/he has the commitment, drive and skills to support implementation. Moreover, effective implementers build supportive networks (the team around the implementer), both formal (eg, steering groups; staff mindfulness groups) and informal (eg, peer support). When there were different implementers, with a variety of seniority and skills, all working towards the same agenda, implementation was more effective. The fit between the implementer and the service context (eg, mental health policies, health service priorities and working culture) is key. Over the implementation journey, there was typically a starting point, which built momentum through bottom-up, grass-roots efforts from a single or small set of implementers, with the prevailing conditions being key to how easy it was for these implementers to build up momentum.

There was evidence that the model could explain substantive progress or stalling out of implementation (figure 1), particularly where we found that implementation journeys typically had one or more pivot points. Going forward, successful implementation is likely to be supported by using this explanatory model to plan, execute and evaluate progress in implementation. Pivot points involved the coming together of conditions for implementation to take a substantive leap forwards or to stall, and in some cases stall out. The pivot points varied from case to case, but they were typically characterised by an alignment of skilful implementation efforts with the context for implementation (figure 2). Some examples of upward pivot points were the creation of an MBCT centre of excellence, with attention to governance, training, research and staff well-being; securing support and resources for service provision; integration with staff well-being agendas; securing internal training and supervision and integration with regional and national depression programmes. Conversely, examples of downward pivot points were resources being withdrawn, implementers leaving, service reorganisations and changes in service priorities and culture. Effective implementation involves proactive engagement with both these downward pivot points and the upward pivot points. Indeed, in this framing, the distinction between ‘barriers and facilitators’ is very blurred, and more down to how people with different perspectives express them—many barriers are absence of facilitators (eg, adequate resources) and vice versa. Successful implementation involves awareness of how the team of implementers, MBCT intervention and context need to be continually adapted, fine-tuned and aligned for success, and using this awareness, particularly at key pivot points to facilitate sustainable implementation.

A metaphor of *evolutionary fitness* helps explain how implementation can develop and strengthen over time, through ensuring that adaptations build ‘fitness’. Where implementers continually assessed the needs of the service context, evaluated MBCT’s outcomes and adapted MBCT accordingly, it was more likely to evolve successfully.

In particular, services’ long-term sustainability was characterised by investment in training and supervision. This created the capacity for expansion and succession planning. Since this research, an MBCT Training Pathway international consensus statement has been published.^44^ This framework alongside UK Good Practice Guidance for Training and Supervision was used by NHS England as the basis for the development of a national MBCT training curriculum. In line with the recommendations of the Mindfulness All Party Parliamentary Group,^18^ Health Education England has now commissioned the training of MBCT teachers within the Improving Access to Psychological Therapies workforce in England.

### Limitations

We sampled from a wide range of stakeholders and study sites. Data collection and analysis were theoretically driven and systematically conducted, including the perspectives of the research team, service user groups and key stakeholder groups. We have developed metaphors and resources to inform implementation guidance.^19 43^

Sampling led to those most invested in MBCT implementation being over-represented because they were able to give fuller accounts of a service’s implementation history. Moreover, the research team itself has interest and affiliations both to the MBCT intervention and to the PARIHS conceptual framework. We tried to balance this with purposively including broader constituencies, including sceptics, using our PPI group and study advisory group to provide checks and balances and through declaring our interests. We also obtained both positive and negative accounts suggesting that those invested in MBCT implementation provided balanced accounts. As best we could, we used a systematic and transparent approach to articulating our questions, methods^23^ and analysis. Effective implementation needs to consider sustainability at every stage, and given the relative paucity of examples of MBCT implementation over many years to date, the data were thin with regard to sustainability beyond 5–10 years. Finally, we speculate that the proposed framework would be potentially translatable to other countries, but with differences of emphasis (eg, contextual national mental health policies, and resources allocated to mental health). Interventional studies to test whether this model is translatable and can support both effective and sustainable implementation would be an obvious next step. For example, we could envisage a cluster RCT design of an implementation based on this explanatory model versus implementation as usual, with sustainability of implementation (ie, improved patient access to MBCT over a 5–10 years period) as the primary outcome.

## Conclusions

This study is one of the largest and most systematic explorations of the implementation of a psychological therapy. Although access to MBCT across the UK has improved since our 2011 survey,^45^ it remains patchy and variable. Through several phases of work, we developed themes that describe what facilitates MBCT implementation and a theoretical framework of how MBCT becomes sustainably embedded. The ‘long view’ for sustainable implementation suggests that implementation is a journey over many years, which requires clarity of intention, persistent individual and organisational commitment, and an ongoing evolution as both the context and the intervention change and adapt. We have used this research to develop an online resource to support sustainable implementation in health services (http://www.implementing-mbct.com/).^43^
